# Arrhythmogenic drugs can amplify spatial heterogeneities in the electrical restitution in perfused guinea-pig heart: An evidence from assessments of monophasic action potential durations and JT intervals

**DOI:** 10.1371/journal.pone.0191514

**Published:** 2018-01-19

**Authors:** Oleg E. Osadchii

**Affiliations:** 1 Department of Biomedical Sciences, University of Copenhagen, Copenhagen, Denmark; 2 Department of Health Science and Technology, University of Aalborg, Aalborg, Denmark; University of Minnesota, UNITED STATES

## Abstract

Non-uniform shortening of the action potential duration (APD_90_) in different myocardial regions upon heart rate acceleration can set abnormal repolarization gradients and promote arrhythmia. This study examined whether spatial heterogeneities in APD_90_ restitution can be amplified by drugs with clinically proved proarrhythmic potential (dofetilide, quinidine, procainamide, and flecainide) and, if so, whether these effects can translate to the appropriate changes of the ECG metrics of ventricular repolarization, such as JT intervals. In isolated, perfused guinea-pig heart preparations, monophasic action potentials and volume-conducted ECG were recorded at progressively increased pacing rates. The APD_90_ measured at distinct ventricular sites, as well as the JTpeak and JTend values were plotted as a function of preceding diastolic interval, and the maximum slopes of the restitution curves were determined at baseline and upon drug administration. Dofetilide, quinidine, and procainamide reverse rate-dependently prolonged APD_90_ and steepened the restitution curve, with effects being greater at the endocardium than epicardium, and in the right ventricular (RV) vs. the left ventricular (LV) chamber. The restitution slope was increased to a greater extent for the JTend vs. the JTpeak interval. In contrast, flecainide reduced the APD_90_ restitution slope at LV epicardium without producing effect at LV endocardium and RV epicardium, and reduced the JTpeak restitution slope without changing the JTend restitution. Nevertheless, with all agents, these effects translated to the amplified epicardial-to-endocardial and the LV-to-RV non-uniformities in APD_90_ restitution, paralleled by the increased JTend vs. JTpeak difference in the restitution slope. In summary, these findings suggest that arrhythmic drug profiles are partly attributable to the accentuated regional heterogeneities in APD_90_ restitution, which can be indirectly determined through ECG assessments of the JTend vs. JTpeak dynamics at variable pacing rates.

## Introduction

In susceptible patients, antiarrhythmic drug therapies can paradoxically precipitate malignant ventricular tachyarrhythmia, torsade de pointes, thereby increasing mortality rates [1]. The proarrhythmic responses most frequently occur with class Ia and Ic Na^+^ channel blockers, and class III agents that block *I*_Kr_, the rapid component of the delayed rectifier. For instance, the DIAMOND clinical trial has raised serious concerns regarding torsadogenic effects produced by dofetilide, class III antiarrhythmic agent, in patients with congestive heart failure [2]. In the CAST trial, an increase in sudden arrhythmic death was observed upon administration of flecainide, class Ic Na^+^ channel blocker, in patients with healed myocardial infarction [3]. An increased occurrence of torsade de pointes has also been reported in studies that utilized class Ia agents, quinidine and procainamide [4–5].

Drug-induced cardiac electrical instability often develops unexpectedly, and its early detection is difficult due to the limited prognostic value of the existing arrhythmic biomarkers. Recently, the electrical restitution analysis has been proposed as a novel approach for evaluation of the drug safety profile [6–7]. When ventricular action potential duration (APD) is plotted vs. the diastolic interval, arrhythmic effects can be predicted via two modifications, namely, the increased maximum slope of the APD restitution curve, and the amplified dispersion of the restitution slope value determined at different ventricular recording sites. Steep electrical restitution precipitates beat-to-beat oscillations in APD (electrical alternans) during tachycardia, whereas non-uniform APD shortening at distinct myocardial regions sets abnormal spatial repolarization gradients; both mechanisms, therefore, can facilitate localized conduction block leading to the wavebreak and ventricular fibrillation.

Although drug-induced arrhythmia was partly attributed to the steepening of the electrical restitution in some studies [8–12], the putative contribution of the amplified spatial heterogeneities in restitution kinetics to the arrhythmic drug profile has not been examined yet. Likewise, it remains uncertain as to whether modified spatial heterogeneities of the APD restitution upon drug administration can translate to appropriate changes of the ECG metrics of ventricular repolarization, such as JT intervals. The JTpeak and the JTend intervals are thought to reflect the shortest and the longest action potential duration, respectively, when repolarization is assessed at multiple recording sites across LV wall [13], or along the transmural and apicobasal axes in both ventricular chambers [14–17]. Therefore, it can be hypothesized that the difference in the maximum slope of the JTpeak vs. JTend restitution would roughly approximate ventricular site-dependent variations of the APD restitution slope value, upon drug infusions.

With these considerations in mind, the present study examined effects of four pharmacological agents with clinically proved arrhythmogenic effects (dofetilide, quinidine, procainamide, and flecainide) on the spatial heterogeneities in APD restitution and the JTpeak vs. JTend difference in the restitution slope, in isolated, perfused guinea-pig hearts.

## Materials and methods

This study complies with the European Community Guidelines for the Care and Use of Experimental Animals, and was approved by the Animal Ethics Screening Committee of the Panum Institute (clearance number: 2010/561-1799).

### Isolated, Langendorff-perfused heart preparations

The heart preparations were obtained from female Dunkin-Hartley guinea-pigs, assuming that females have reduced cardiac repolarization reserve and therefore more susceptible to drug-induced proarrhythmia, as compared to males [18]. The experiments on isolated, perfused hearts were performed as described previously [19–20]. The guinea-pigs (body weight 400–500 g) were anesthetized with sodium pentobarbital (50 mg/kg i.p.) and anticoagulated with heparin (1000 IU/kg i.p.). The chest was opened, the hearts were immediately excised, mounted on a Langendorff perfusion set-up (Hugo Sachs Elektronik-Harvard Apparatus GmbH, March-Hugstetten, Germany) and perfused via the aorta at a constant flow (15 ml/min) with carefully filtered, warmed physiological saline solution saturated with 95%O_2_ and 5%CO_2_. The perfusion solution contained (in mM) 118.0 NaCl; 4.7 KCl; 2.5 CaCl_2_; 25 NaHCO_3_; 1.2 KH_2_PO_4_; 1.2 MgSO_4_; and 10.0 glucose, and had a pH of 7.4. The aortic perfusion pressure (65–70 mm Hg) was measured with a ISOTEC pressure transducer and the coronary flow rate was determined using an ultrasonic flowmeter probe (Transonic Systems Inc., USA) placed just above the aortic cannula. The electrical activity of the heart preparations was assessed from the volume-conducted ECG as well as monophasic action potential recordings. Throughout the experiments, the heart preparations were kept immersed in the temperature-controlled, perfusate-filled chamber to minimize thermal loss. Aortic pressure, coronary flow rate, ECG and monophasic action potentials were continuously monitored using the 16-channel PowerLab system (ADInstruments, Oxford, UK).

### Electrophysiological recordings

**Fig 1:**
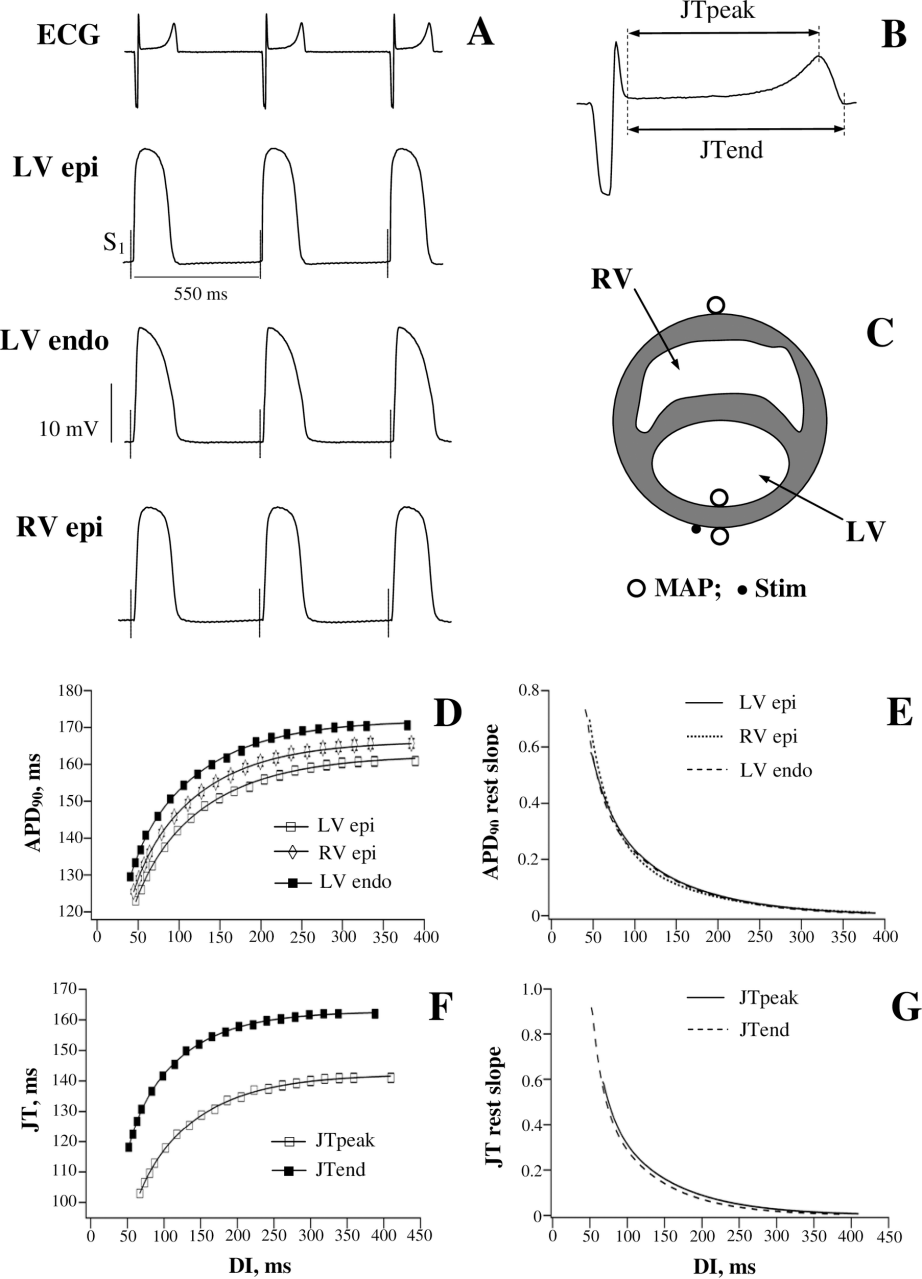
Basal electrophysiological recordings and the restitution of ventricular action potential duration and JT intervals. Panel A shows representative ECG and monophasic action potentials (MAP) recorded at the left ventricular (LV) epicardium (epi), LV endocardium (endo), and the right ventricular (RV) epicardium upon S_1_-S_1_ pacing at a cycle length of 550 ms. The moments of S_1_ application are shown by vertical dotted lines on the MAP traces. The first beat from ECG recording in panel A is shown at a larger scale on panel B in order to illustrate the measurements of the JTpeak and JTend intervals. Panel C shows location of the MAP recording electrodes (open circles), and the LV stimulating (Stim) electrode (filled circle). Panels D and F show averaged action potential durations (APD_90_) measured at distinct ventricular regions (panel D), and the JTpeak and JTend intervals determined from ECG (panel F) during cardiac pacing at variable diastolic intervals (DI) prior to drug infusion in 30 experiments. The slope values obtained from the APD_90_ and JT restitution (rest) curves are plotted in panels E and G, respectively.

In order to slow the intrinsic beating rate and enable ventricular pacing over a wide range of diastolic intervals, both atria were removed and the atrioventricular (AV) node was crushed mechanically with forceps prior to taking electrophysiological recordings. Monophasic action potentials (MAP) were obtained from the endocardial and the opposite epicardial recording sites at the base of the left ventricular (LV) lateral wall, and from the right ventricular (RV) epicardial base (Fig 1, panels A and C) using spring-loaded pressure contact electrodes (Hugo Sachs Electronik-Harvard Apparatus GmbH, March-Hugstetten, Germany). The MAP duration was measured at 90% repolarization (APD_90_). Electrical stimulations were applied at LV epicardium close to the adjacent MAP recording electrode using 2 ms rectangular pulses of twice diastolic threshold current generated by a programmable stimulator (Hugo Sachs Electronik-Harvard Apparatus GmbH, March-Hugstetten, Germany). LV pacing thresholds were measured both at baseline and upon drug infusion, and the stimulating current strength was adjusted appropriately whenever necessary.

On the volume-conducted ECG, JT intervals were measured from the end of the QRS complex (J point) to the peak of the T wave (JTpeak interval), and to the end of the T wave (JTend interval) (Fig 1, panel B).

### Electrical restitution

The electrical restitution kinetics was assessed using the steady-state pacing protocol [10–12]. In brief, a train of 50 pulses was applied, and the MAP duration at distinct ventricular recording sites, as well as JTpeak and JTend intervals on ECG were determined in the last beat in a train, while progressively decreasing the S_1_-S_1_ pacing interval in successive stimulations. The measurements were started with a S_1_-S_1_ cycle length of 550 ms (the longest pacing interval producing no ventricular escape beats), which was then reduced to 500 ms, followed by further reductions in steps of 20 ms over an S_1_-S_1_ range from 500 to 200 ms, and by 5–10 ms reductions from 200 ms down to the pacing intervals (about 170 ms) producing 2:1 conduction block. In AV-blocked preparations, the minimum pacing intervals achieved with this protocol remain significantly greater than the threshold value of S_1_-S_1_ interval for inducing repolarization alternans (90–100 ms in intact guinea-pig heart) [21], meaning that the whole pacing protocol could have been completed without inducing cardiac electrical instability.

Once the electrical stimulations were completed, appropriate diastolic intervals (DI) were calculated as a difference between the S_1_-S_1_ cycle length and the measured APD_90_ (APD_90_ restitution) or JTpeak and JTend interval (JT restitution) values. The electrical restitution was analysed by plotting APD_90_ and JT intervals as a function of the preceding DI. The restitution curves were fitted using double-exponential function: y = y_0_ + A_1_exp^(-DI/τ1)^ + A_2_exp^(-DI/τ2)^, where y represents APD_90_ or JT interval value, y_0_ is a free-fitting variable, A_1_ and A_2_ are the amplitudes, and τ_1_ and τ_2_ are the time constants of the fast (A_1_ and τ_1_) and slow (A_2_ and τ_2_) exponential components obtained by a least squares fit. The curve fitting was performed using Igor Pro 6.0 software (WaveMetrics, Inc., Portland, OR, USA).

The obtained exponential curves were differentiated to determine the maximum restitution slope value (Fig 1, panels D-G). Thereafter, spatial dispersion in APD_90_ restitution kinetics was assessed by calculating the difference between the maximum restitution slopes measured at LV endocardium vs. LV epicardium, and those measured at RV epicardium vs. LV epicardium. With ECG recordings, the difference between the maximum slopes obtained from JTend vs. JTpeak restitution curves was taken as an ECG estimate of the regional ventricular heterogeneities in electrical restitution.

### Drug infusions

In total, 30 heart preparations were used in this study, in order to examine effects produced by dofetilide, quinidine, procainamide, and flecainide (7–8 experiments in each study group). For precise dosing, drug infusions were performed at a rate of 0.3 ml/min using a calibrated infusion pump, while perfusing the hearts with protein-free saline solution at a constant coronary flow rate (see above). Dofetilide (10 nM), quinidine (5 μM), procainamide (10 μM), and flecainide (1.5 μM) (all from Sigma-Aldrich, Germany) were infused over 30 min, at concentrations close to the maximum free (i.e. protein-unbound) therapeutic plasma levels [22–25] (S1 Table).

### Data analysis

Data are expressed as mean ± standard error of the mean. One-way ANOVA was used for multiple comparisons, and paired t-tests were used to compare two data sets. *P* values less than 0.05 were considered to be significant.

## Results

### Restitution of APD_90_ and JT intervals in basal conditions

Fig 1 shows representative ECG and monophasic action potential recordings (panel A), and the summary data for the restitution of ventricular APD_90_ (panels D and E) and JT intervals (panels F and G) obtained prior to drug infusion in heart preparations from all experimental groups. Over a range of variable diastolic intervals, action potential duration was found to be 5–10 ms shorter at LV epicardium compared to the corresponding LV endocardial or RV epicardial APD_90_ values (Fig 1, panel D). On the ECG, JTpeak interval was about 20 ms shorter than JTend interval (Fig 1, panel F). A decrease in LV pacing cycle length from 550 ms to 168±2 ms (the minimum S_1_-S_1_ interval with preserved LV capture) provoked an exponential reduction of both ventricular APD_90_ and JT intervals, whereby the steepness of the electrical restitution curves was progressively increasing upon a reduction of the diastolic interval (Fig 1, panels E and G). Importantly, the maximum slope of APD_90_ restitution, attained at the shortest DI, was greater at LV endocardium (0.73±0.03) and RV epicardium (0.69±0.03) compared to the slope determined at LV epicardium (0.57±0.02) (Fig 1, panel E; *P* = 0.0001), indicating the presence of spatial heterogeneities in electrical restitution, both over LV transmural and RV-to-LV transepicardial planes. With ECG recordings, the maximum restitution slope was greater for JTend interval (0.91±0.05) compared to JTpeak interval (0.58±0.02) (Fig 1, panel G; *P*<0.0001), thus providing an ECG correlate for assessments of the regional non-uniformities in electrical restitution.

### Dofetilide

#### APD_90_ restitution

**Fig 2:**
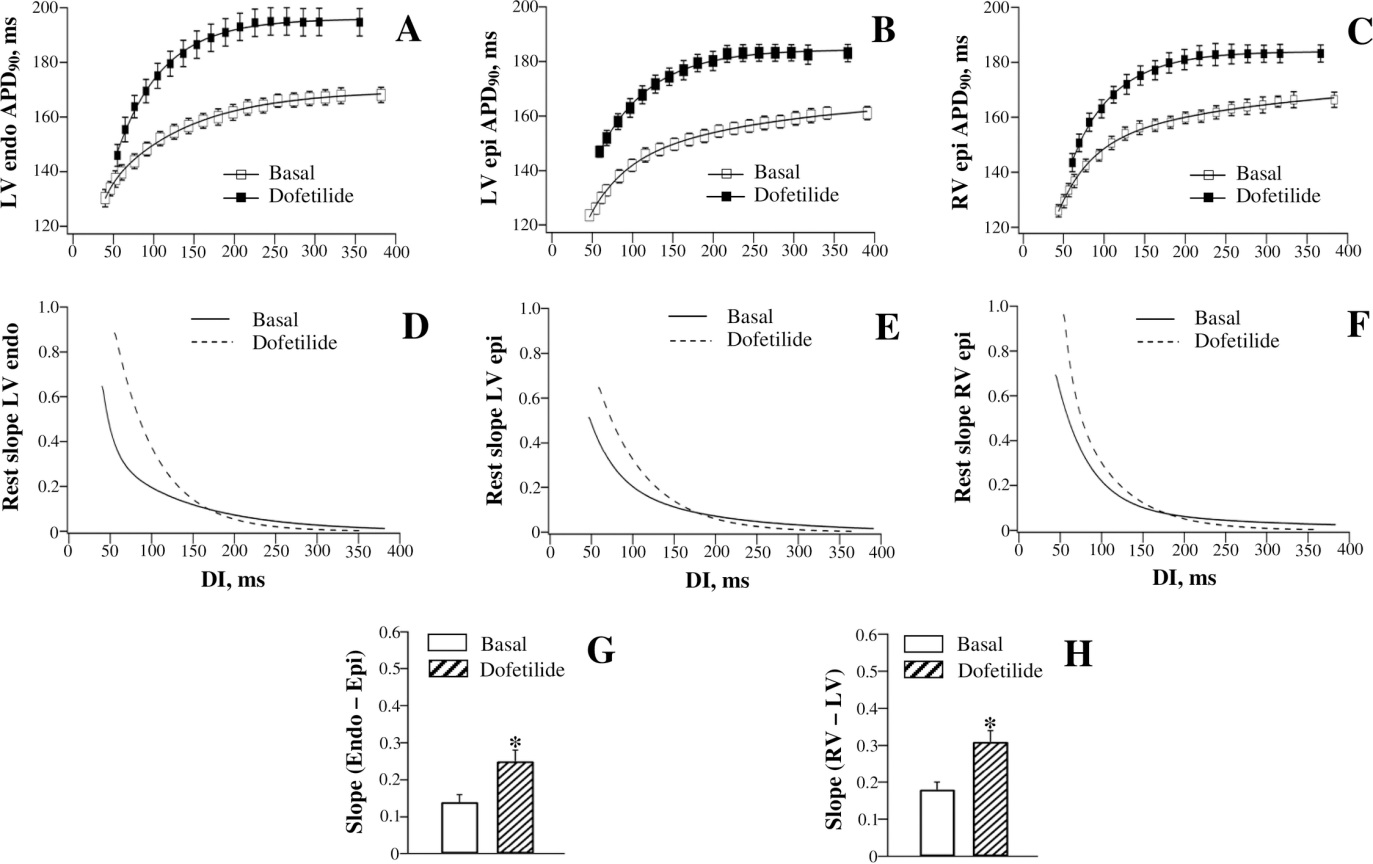
Effects of dofetilide on the restitution of action potential duration assessed at distinct ventricular recording sites. Action potential duration (APD_90_) was measured at baseline and upon drug infusion at the left ventricular (LV) endocardium (endo) (panel A), LV epicardium (epi) (panel B), and the right ventricular (RV) epicardium (panel C), and then plotted as a function of preceding diastolic interval (DI). The APD_90_ restitution (rest) curves were differentiated in order to determine the maximum restitution slope value attained at the shortest DI (panels D, E, and F). Panel G shows the difference in the maximum restitution slope determined at LV endocardium vs. LV epicardium. Panel H shows the difference in the maximum restitution slope determined at RV vs. LV epicardium. *P<0.05 vs. basal value (in panels G and H).

Fig 2 shows dofetilide effects on APD_90_-to-DI relations assessed at different ventricular regions. Dofetilide significantly prolonged repolarization at LV endocardium, LV epicardium, and RV epicardium, thus causing the APD_90_ restitution curves to shift upwards (Fig 2, panels A, B, and C). An increase in APD_90_ by dofetilide was markedly accentuated at the long (above 250 ms) as compared to the short (less than 100 ms) diastolic intervals, which contributed to the steepening of the restitution curve, as evidenced by increased maximum slope values, at each ventricular recording site (Fig 2, panels D, E, and F).

The reverse rate-dependent effect of dofetilide was quantified by calculating the difference in APD_90_ increase obtained at the maximum as compared to the minimum diastolic intervals. For example, at LV endocardium (Fig 2, panel A), dofetilide prolonged APD_90_ from 168±3 ms to 195±5 ms at the maximum DI (~380 ms), while increasing APD_90_ from 140±3 ms to 146±4 ms at the minimum DI (~50 ms). The difference in APD_90_ lengthening produced by dofetilide at the maximum DI vs. the minimum DI (27 ms vs. 6 ms) therefore amounted 21±2 ms. At LV epicardium (Fig 2, panel B), the difference in drug-induced APD_90_ lengthening determined at the maximum DI vs. the minimum DI (22 ms vs. 14 ms) was found to be only 8±1 ms, whereas at RV epicardium (Fig 2, panel C) this difference (18 ms vs. 6 ms) amounted 12±1 ms.

In connection with the spatial variations in the reverse rate-dependent effect on APD_90_, dofetilide produced a greater increase in the maximum slope of electrical restitution at LV endocardium (from 0.65±0.06 to 0.90±0.08) compared to LV epicardium (from 0.51±0.05 to 0.65±0.06) (Fig 2, panels D and E). As a result, the endocardial-to-epicardial difference in APD_90_ restitution slope determined at baseline was further amplified by dofetilide (Fig 2, panel G). Likewise, a greater steepening of the APD_90_ restitution curve by dofetilide was produced at RV epicardium (the maximum slope increased from 0.69±0.06 to 0.96±0.09) compared to LV epicardium (Fig 2, panels E and F), thus increasing the RV-to-LV dispersion in the restitution slope value (Fig 2, panel H).

#### JT restitution

**Fig 3:**
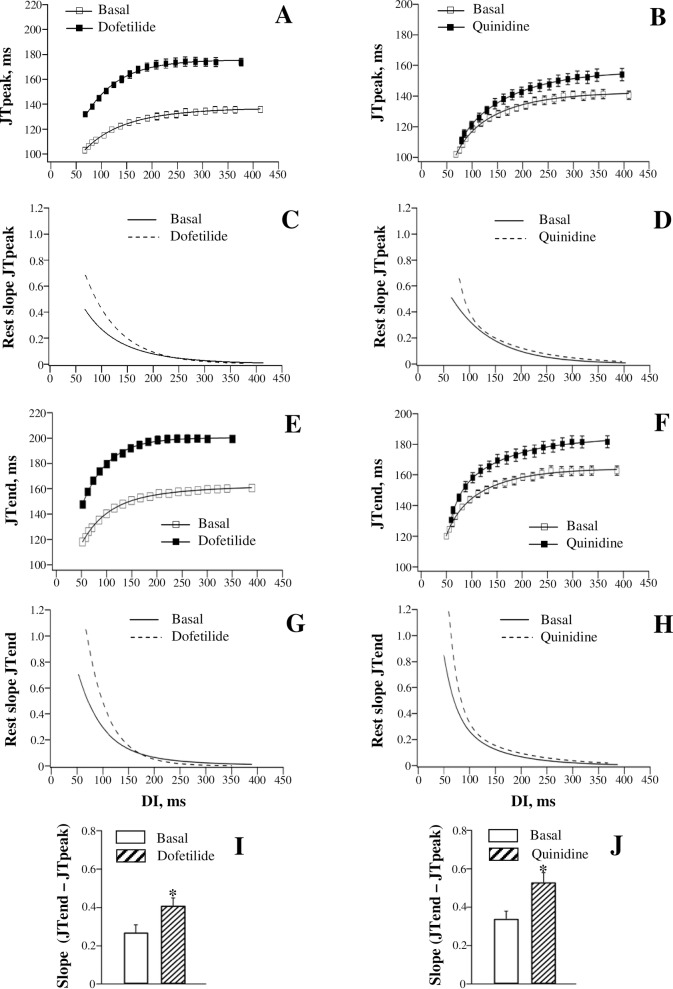
Effects of dofetilide and quinidine on the restitution of JTpeak and JTend intervals. JTpeak (panels A and B) and JTend (panels E and F) intervals were measured on ECG at variable pacing rates, both at baseline and upon drug infusion, and plotted as a function of preceding diastolic interval (DI). The JTpeak and JTend restitution (rest) curves were differentiated in order to determine the maximum restitution slope value attained at the shortest DI (panels C, D, G, and H). Drug effects on the JTpeak vs. JTend difference in the maximum restitution slope are shown in panel I (dofetilide) and panel J (quinidine). *P<0.05 vs. basal value (in panels I and J).

Fig 3 (the left set of panels) shows dofetilide effects on the dynamics of JT intervals at variable pacing rates. Dofetilide prolonged the JTpeak and JTend intervals in the reverse rate-dependent manner (Fig 3, panels A and E), and increased the steepness of the JTpeak and JTend restitution curves (Fig 3, panels C and G). The JTpeak interval (Fig 3, panel A) was prolonged at the maximum DI by 36 ms (from 136±2 ms to 172±3 ms), and at the minimum DI by 29 ms (from 103±2 ms to 132±2 ms). The JTend interval (Fig 3, panel E), was prolonged at the maximum DI by 39 ms (from 160±2 ms to 199±3 ms), and at the minimum DI by 26 ms (from 119±2 ms to 145±2 ms). The difference in JT prolongation obtained at the maximum DI vs. the minimum DI was larger with JTend (13±1 ms) compared to JTpeak interval (7±1 ms). Accordingly, a greater increase in the maximum restitution slope by dofetilide was observed with JTend interval (from 0.70±0.06 to 1.08±0.09) compared to JTpeak interval (from 0.42±0.04 to 0.68±0.06) (Fig 3, panels C and G). These changes contributed to significantly increased JTend vs. JTpeak difference in the maximum restitution slope value upon dofetilide administration (Fig 3, panel I).

### Quinidine and procainamide

#### APD_90_ restitution

**Fig 4:**
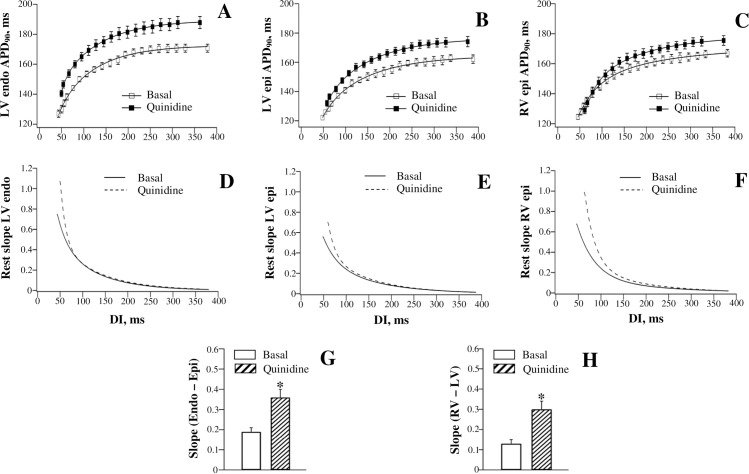
Effects of quinidine on the restitution of action potential duration assessed at distinct ventricular recording sites.

**Fig 5:**
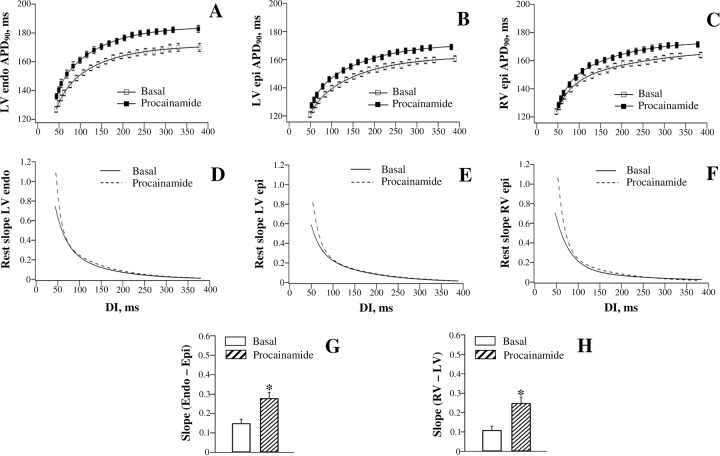
Effects of procainamide on the restitution of action potential duration assessed at distinct ventricular recording sites.

Figs 4 and 5 show effects of quinidine and procainamide, respectively, on APD_90_-to-DI relations assessed at different ventricular regions. Both agents produced a reverse rate-dependent prolongation of ventricular repolarization, and modulated APD_90_ restitution in the same way as dofetilide. With quinidine (Fig 4, panels A, B, and C), the difference in APD_90_ change obtained at the maximum DI vs. the minimum DI was found to be larger at LV endocardium (12±1 ms) and RV epicardium (11±1 ms) compared to that determined at LV epicardium (8±1 ms). With procainamide (Fig 5, panels A, B, and C), the corresponding values amounted 9±1 ms (LV endocardium), 7±1 ms (RV epicardium), and 4±1 ms (LV epicardium). Ventricular site-dependent variations in drug effects on APD_90_ translated to a greater steepening of the electrical restitution curve at LV endocardium than at LV epicardium, and in RV compared to LV chamber, upon infusion of quinidine and procainamide (panels D, E, and F in Figs 4 and 5). Consequently, both LV endocardial-to-epicardial and RV-to-LV epicardial dispersion of the maximum APD_90_ restitution slope was increased by these agents (panels G and H in Figs 4 and 5).

#### JT restitution

**Fig 6:**
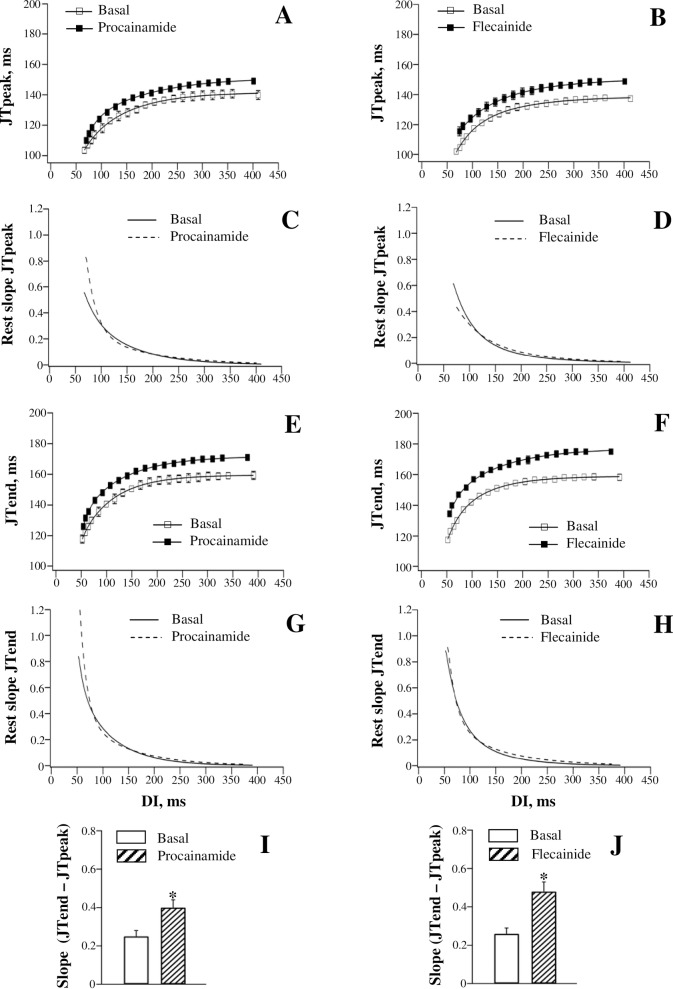
Effects of procainamide and flecainide on the restitution of JTpeak and JTend intervals.

Fig 3 (the right set of panels) and Fig 6 (the left set of panels) show effects of quinidine and procainamide, respectively, on the restitution of JT intervals. Quinidine and procainamide reverse rate-dependently prolonged the JTpeak and JTend intervals, and increased the steepness of the JTpeak and JTend restitution curves. With both agents, the difference in the relative JTend change obtained at the maximum DI vs. the minimum DI (Quinidine: 18±2 ms; Procainamide: 10±1 ms) was found to exceed the corresponding difference in JTpeak change (Quinidine: 11±1 ms; Procainamide: 6±1 ms). Accordingly, a greater steepening of the restitution curve was observed with JTend compared to JTpeak intervals upon infusion of quinidine (Fig 3, panels D and H) and procainamide (Fig 6, panels C and G). These disproportional changes resulted in the amplified JTend vs. JTpeak dispersion of the maximum restitution slope upon administration of quinidine (Fig 3, panel J) and procainamide (Fig 6, panel I).

### Flecainide

#### APD_90_ restitution

**Fig 7:**
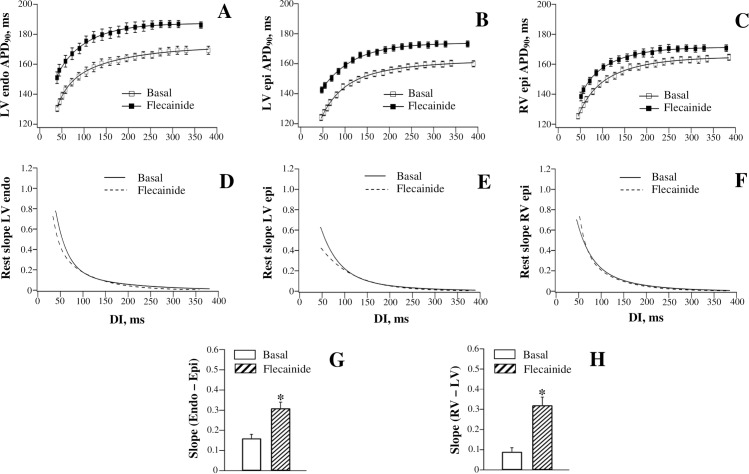
Effects of flecainide on the restitution of action potential duration assessed at distinct ventricular recording sites.

Fig 7 shows flecainide effects on APD_90_-to-DI relations determined at the three ventricular recording sites. Flecainide produced a comparable increase in APD_90_ at the maximum DI vs. the minimum DI, when assessed at LV endocardium and RV epicardium. For example, at LV endocardium (Fig 7, panel A), APD_90_ was prolonged by 17 ms (from 169±3 ms to 186±3 ms) at the maximum DI, and by 19 ms (from 131±2 ms to 150±3 ms) at the minimum DI. At RV epicardium (Fig 7, panel C), the corresponding APD_90_ increase amounted 6 ms at the maximum DI, and 8 ms at the minimum DI. These effects resulted in no change in the maximum APD_90_ restitution slope determined at LV endocardium (Basal: 0.73±0.06; Flecainide: 0.78±0.06) and RV epicardium (Basal: 0.71±0.06; Flecainide: 0.74±0.06) (Fig 7, panels D and F). In contrast, at LV epicardium (Fig 7, panel B), the relative increase in APD_90_ produced by flecainide at the minimum DI (19 ms, from 124±2 ms to 143±2 ms) was more prominent than that produced at the maximum DI (13 ms, from 160±2 ms to 173±2 ms). This resulted in flattening of the restitution curve obtained at LV epicardium (Fig 7, panel E), as evidenced by a reduction in the maximum slope from 0.62±0.05 to 0.42±0.04 (*P* = 0.01). No change in the maximum restitution slope determined at LV endocardium and RV epicardium, in association with the reduced slope value at LV epicardium, translated to the increased spatial heterogeneities in electrical restitution upon flecainide administration, both over LV transmural and RV-to-LV epicardial planes (Fig 7, panels G and H, respectively).

#### JT restitution

Fig 6 (the right set of panels) shows flecainide effects on the restitution of JT intervals. Flecainide prolonged JTend interval (Fig 6, panel F) by 17 ms, both at the maximum DI (JTend change: from 158±2 ms to 175±1 ms) and at the minimum DI (JTend change: from 118±1 ms to 135±1 ms). The uniform increase in JTend interval at variable pacing rates translated to no drug effect on the maximum slope of the JTend restitution curve (Basal: 0.87±0.07; Flecainide: 0.90±0.07) (Fig 6, panel H). In contrast, an increase in JTpeak interval (Fig 6, panel B) produced by flecainide tended to be greater at the minimum DI (14 ms, from 102±1 ms to 116±3 ms) compared to the maximum DI (10 ms, from 138±1 ms to 148±1 ms), thus causing the JTpeak restitution curve to flatten (Fig 6, panel D). The maximum JTpeak restitution slope was reduced from 0.61±0.05 to 0.43±0.04 (*P* = 0.01) by flecainide. No change in JTend restitution kinetics, in association with reduced JTpeak restitution slope, resulted in amplified JTend vs. JTpeak difference in the maximum restitution slope value (Fig 6, panel J).

## Discussion

The main findings from the present study are twofold. First, it is shown that LV transmural and RV-to-LV epicardial dispersion of the maximum APD_90_ restitution slope is amplified by drugs that reportedly produce arrhythmic responses in the clinical setting (dofetilide, quinidine, procainamide, and flecainide). Second, the study demonstrates that an increase in regional APD_90_ restitution heterogeneities translates to the increased JTpeak vs. JTend difference in the maximum restitution slopes determined from ECG recordings at variable pacing rates.

### Electrical restitution and arrhythmogenesis

Electrical restitution refers to the rate-dependent shortening of ventricular repolarization, which is attributed to incomplete deactivation of *I*_Kr_ and *I*_Ks_ (the rapid and the slow components of the delayed rectifier, respectively), and to the increased intracellular Na^+^ and Ca^2+^ concentrations at rapid cardiac activation rates [26–27]. The latter changes can decrease APD via effects on the Na^+^-Ca^2+^ exchanger and Na^+^-K^+^ pump, and through the Ca^2+^-induced inactivation of *I*_Ca_ in cardiac myocytes.

A steep slope of APD rate adaptation has been shown to facilitate ventricular fibrillation by inducing APD and Ca^2+^ transient alternans in cardiac myocytes [6–7, 28]. The clinical relevance of this mechanism, nevertheless, was challenged in studies that demonstrate no difference in the maximum APD restitution slope determined in patients with structural heart disease susceptible to arrhythmia compared to the control subjects [29–30]. Likewise, the maximum restitution slope reportedly has no prognostic value at predicting arrhythmic death over long-term follow-up [29, 31]. These findings therefore suggest the importance of restitution parameters other than the maximum slope in the mechanisms of arrhythmogenesis.

Owing to the spatial heterogeneities in expression and function of repolarizing ionic currents [32], the amount of the rate-dependent APD shortening is variable at distinct ventricular regions [33–34]. In the human heart, the APD restitution slopes are distributed non-uniformly throughout both the epicardial and endocardial surface, and there is a considerable LV-to-RV difference in the averaged steepness of the restitution curve [35–36]. Across LV wall, the APD restitution slope is steeper at midmyocardium compared to subepicardial and subendocardial regions in non-failing human hearts [37]. Spatial non-uniformities in electrical restitution are markedly accentuated in patients with inducible tachyarrhythmia compared to those without arrhythmia [30, 38], and in experimental studies, they can be amplified by proarrhythmic interventions such as sympathetic neural stimulation [39] and global ischemia [40]. Following a sudden reduction of the diastolic interval, for example, imposed by a premature ectopic activation, a greater APD shortening at the ventricular site with a steeper restitution slope compared to the adjacent site with a shallower slope can contribute to the reversal of the local repolarization gradient, and provide a substrate for re-entry [33, 41]. In this regard, there is a strong evidence to demonstrate that a wavebreak precipitating sustained arrhythmia can occur in the absence of steepened APD restitution, providing that spatial dispersion of the restitution slope exceeds a certain critical level [34, 42]. These studies therefore raise the point that amplified regional restitution heterogeneities represent an independent arrhythmic determinant that should be considered in assessments of the drug safety profile.

### Basal spatial restitution heterogeneities in guinea-pig heart

The present study reports on significant regional restitution heterogeneities in guinea-pig heart, wherein the maximum slope of APD restitution is greater at the endocardium vs. the epicardium, and in the RV vs. the LV chamber (Fig 1, panels D and E). These results are likely accounted for by the site-dependent variations in density of the outward K^+^ currents that govern phase-3 repolarization. In guinea-pig heart, the density of *I*_K_, the delayed rectifier, is significantly less in endocardial than epicardial myocytes [43], and *I*_K1_ current (the inward rectifier) is smaller in RV than LV myocytes [44]. Importantly, the slow component of *I*_K_ (*I*_Ks_) is reduced to a greater extent than the rapid component (*I*_Kr_), in endocardium vs. epicardium [43], indicating a greater *I*_Kr_-to-*I*_Ks_ ratio in endocardial cells. In connection with this, the simulations that utilize the guinea-pig ventricular cell model suggest that a larger *I*_Kr_-to-*I*_Ks_ ratio contributes to the faster rate of APD changes at short diastolic intervals [26, 45], thus providing a mechanism for the regional differences in restitution kinetics, at least over transmural plane.

The recent experimental works increasingly recognize that the steady-state action potential duration is the principal intrinsic determinant of the restitution slope; a greater basal APD independently contributes to steepening of the restitution curve [33, 46]. The results from the present study partly concur with this notion, as the LV endocardium and RV epicardium were found to exhibit both a longer steady-state APD and a steeper maximum restitution slope, when compared to those determined at the LV epicardium (Fig 1, panels D and E).

### Drug effects

In this study, dofetilide, quinidine, and procainamide prolonged APD to a greater extent at the long as compared to the short DIs, and therefore steepened the restitution curve (panels A-F in Figs 2, 4 and 5). The reverse rate-dependent prolongation of APD is an important attribute of the drug-induced *I*_Kr_ block [47–48], which is relevant to these agents—dofetilide is a specific *I*_Kr_ blocker [47], whereas quinidine and procainamide, although being categorized as class Ia *I*_Na_ blockers, can significantly reduce *I*_Kr_ in cardiac cells [49–50]. Owing to the regional variations in *I*_Kr_ distribution [32, 43], it can be expected that these drugs would affect the APD restitution in spatially non-uniform manner. Indeed, both the reverse rate-dependent APD lengthening and the resulting increase in the restitution slope by dofetilide, quinidine, and procainamide were found to be greater in endocardium vs. epicardium, and in RV vs. LV chamber, thus contributing to the amplified spatial restitution heterogeneities (panels G and H in Figs 2, 4 and 5).

The prolongation of ventricular repolarization has also been observed with flecainide, the class Ic Na^+^ channel blocker that can inhibit *I*_Kr_ in hERG-expressing cell lines [51] and guinea-pig ventricular myocytes [52]. However, in contrast to dofetilide, quinidine, and procainamide, the APD lengthening by flecainide was not reverse rate-dependent. At LV endocardium and RV epicardium, a proportional APD increase was elicited by flecainide at variable pacing rates, whereas at LV epicardium, the APD was actually prolonged to a greater extent at the short as compared to the long diastolic intervals (Fig 7, panels A-C). As a result, the steepness of the restitution curve was not changed at LV endocardium and RV epicardium, while being reduced at LV epicardium (Fig 7, panels D-F). Hence despite a distinct pattern of APD changes, the flecainide effect on the spatial restitution heterogeneities was similar to those produced by dofetilide, quinidine, and procainamide–both transmural and RV-to-LV dispersion of the maximum restitution slope was accentuated by this agent (Fig 7, panels G and H).

The reason for the contrasting effects of flecainide vs. those produced by the other arrhythmogenic drugs on APD rate-dependency is not clear. Nevertheless, it can be hypothesized that the distinct action of flecainide is partly accounted for by its ability to modify Ca^2+^ handling in cardiac myocytes. Flecainide inhibits the ryanodine receptor-mediated Ca^2+^ release from sarcoplasmic reticulum, which contributes to its therapeutic action in catecholaminergic polymorphic ventricular tachycardia [53–54]. With *I*_Kr_-blocking agents, the reverse rate-dependent APD changes are partly determined by the increased magnitude of *I*_Ks_ at fast pacing rates [47], an effect attributable to both incomplete *I*_Ks_ deactivation, and to the pacing-induced intracellular Ca^2+^ accumulation, which acts to increase *I*_Ks_ conductance [26]. Flecainide, by inhibiting the sarcoplasmic reticulum Ca^2+^ release, can subsequently attenuate the Ca^2+^-dependent increase in *I*_Ks_, and therefore decrease the amount of APD shortening at fast pacing rates. This mechanism may potentially decrease the maximum restitution slope, or at the very least, prevent a steepening of the restitution curve that is expected to result from the concomitant *I*_Kr_-blocking effect of flecainide.

### JT intervals in assessments of electrical restitution

The data suggesting that regional restitution heterogeneities can determine arrhythmic vulnerability regardless of the steepness of the restitution curve [30, 34, 38, 42] stimulated the attempts to develop non-invasive ECG markers that quantify spatial dispersion of the restitution slope [55–56]. The present study expands these efforts by showing that JTpeak vs. JTend difference in the restitution slope can approximate the regional non-uniformities in APD restitution. The assessments were based on measuring the JT rather than the QT intervals, because in the setting of delayed ventricular conduction attributable both to the epicardial pacing and Na^+^ channel blockers effects, a wide QRS complex can significantly contribute to the overall QT duration, thus reducing its specificity in detecting repolarization derangements. Clinically, the risk of incident cardiovascular events is better predicted with measurements of the JT rather than the QT interval [57–59]. It is also noteworthy that in isolated, perfused hearts, the QT interval typically grossly exceeds ventricular action potential duration, and the QT is less sensitive to changes in pacing rate and drug effects, when compared to the corresponding variations in APD_90_ [60–61].

In perfused canine ventricular wedge preparations, the end of epicardial action potential was shown to coincide with the peak of the T wave, whereas full repolarization of the M cells marks the end of the T wave, meaning that the Tpeak-to-Tend interval is a measure of transmural dispersion of repolarization [13]. Conversely, studies that utilized electroanatomic cardiac mapping in open-chest dogs and pigs suggest that the Tpeak-to-Tend interval represents a difference between the earliest and the latest repolarization times determined along all anatomic axes (apicobasal, transmural, interventricular, anterior-posterior) in the whole heart [14–17]. Thus, even though the precise anatomical location of ventricular cells primarily contributing to the Tend vs. those responsible for the Tpeak on ECG is a matter for debate, it appears that JTpeak and JTend intervals roughly correspond to the shortest and the longest action potential durations, respectively, when assessed at multiple ventricular sites. If so, then the dispersion of electrical restitution along the ventricular sites with variable steady-state APD should be closely approximated by the JTpeak vs. JTend difference in the rate adaptation kinetics. In support of this notion, in the present study, the lower value of the maximum restitution slope determined at LV epicardium (0.57±0.02) compared to LV endocardium (0.73±0.03) and RV epicardium (0.69±0.03), was associated with lesser maximum restitution slope for the JTpeak (0.58±0.02) vs. the JTend (0.91±0.05) intervals. Furthermore, the spatial dispersion of APD_90_ restitution and the JTend to JTpeak difference in the restitution slope value were found to increase in parallel upon drug infusions.

#### Clinical implications

Arrhythmogenic drug effects are often precipitated by sudden changes in heart rate, e.g. those provoked by exercise [62], or spontaneous onset and termination of atrial fibrillation [63], raising a possibility that accentuated spatial non-uniformities in APD rate adaptation contribute to arrhythmic substrate. This study provides an evidence in support of this notion by demonstrating that both epicardial-to-endocardial and the LV-to-RV dispersion of the APD_90_ restitution slope is increased by drugs known to have proarrhythmic potential. Furthermore, the association of the JTend vs. JTpeak restitution with appropriate rate-dependent changes in APD_90_ implies that spatial restitution heterogeneities could be assessed indirectly from the surface ECG recordings, for example, during exercise stress tests, thereby providing a non-invasive marker for stratification of arrhythmic risks in the clinical setting.

Importantly, in assessments of arrhythmic susceptibility, the JTpeak vs. JTend restitution would not simply duplicate some other known ECG metrics of repolarization, such as the Tpeak-to-Tend interval. Whilst the Tpeak-to-Tend interval is an estimate of the spatial dispersion of repolarization at a given heart rate, the JT restitution approximates changes in action potential duration obtained over a wide range of cardiac beating rates at the ventricular sites with the earliest (JTpeak) vs. those with the latest (JTend) repolarization times. As such, the JTpeak vs. JTend restitution slope difference is more integrative metric than the Tpeak-to-Tend interval in assessments of repolarization dynamics. This difference can be particularly important in predicting arrhythmia provoked by an abrupt increase in cardiac activation rate. Notably, in the clinical setting, ambulatory ECG monitoring in victims of sudden death suggests that fatal cardiac arrest is often preceded by transient heart rate acceleration [64–65]. In tachycardia, owing to a greater JTend than JTpeak shortening (Fig 1, panel F), the Tpeak-to-Tend interval will be reduced, which may be interpreted as attenuated, rather than increased, arrhythmic susceptibility. In contrast, as the maximum restitution slope is always measured at the shortest diastolic intervals with preserved 1:1 capture, the greatest JTpeak vs. JTend slope difference would be determined at the fast cardiac activation rates, which is consistent with increased arrhythmic tendency in this setting.

#### Limitations

Species-related differences in the outward K^+^ currents that govern cardiac repolarization should be taken into account when considering the practical aspects of this study. In human ventricular myocytes, phase-3 repolarization is mostly controlled by *I*_Kr_, whereas in guinea-pig it is primarily determined by *I*_Ks_ [66–67]. Likewise, *I*_to_, the transient outward K^+^ current, which contributes to phase-1 repolarization in the human ventricular action potential, is not expressed in guinea-pig myocytes [68]. Nevertheless, when considering the electrical restitution kinetics, one argument in support of the guinea-pig model is that the maximum APD restitution slope values determined in this study (Fig 1, panel E) are close to those measured in human patients [35–36], and the spatial non-uniformities in the maximum restitution slope existing either across LV wall or in RV vs. LV sites in the human hearts [35, 37] are also present in the guinea-pig model (Fig 1, panels D and E).

This study explored changes in the rate adaptation of ventricular repolarization upon infusion of arrhythmogenic drugs, with particular focus on APD and JT restitution kinetics. With *I*_Na_ blockers such as flecainide, quinidine, and procainamide, another important proarrhythmic determinant to consider is the restitution of conduction velocity, which determines the excitation wavelength dynamics [69]. Although drug-induced changes in ventricular conduction were not examined in the present study, this aspect was addressed in the previous work [70], which highlighted important differences in class Ia and Ic vs. class Ib Na^+^ channel blockers effects on the restitution of the excitation wavelength.

## Conclusions

Local heterogeneities in distribution of the maximum APD_90_ restitution slope in perfused guinea-pig heart, and the JTpeak vs. JTend difference in the restitution slope value determined from ECG, are increased by drugs with arrhythmogenic profiles, such as dofetilide, quinidine, procainamide, and flecainide.

## Supporting information

S1 Table Therapeutic plasma levels of dofetilide, quinidine, procainamide and flecainide and the drug concentrations used in the present study. (DOC)
