# Supplementary material for: Arrhythmogenic drugs can amplify spatial heterogeneities in the electrical restitution in perfused guinea-pig heart: An evidence from assessments of monophasic action potential durations and JT intervals
Source: PLoS One. 2018 Jan 19;13(1):e0191514. doi: 10.1371/journal.pone.0191514 (PMC5774816; doi:10.1371/journal.pone.0191514)
Supplement: S1 Table — (DOC) [file pone.0191514.s001.doc]

**S1 Table.** Therapeutic plasma levels of dofetilide, quinidine, procainamide and flecainide and the drug concentrations used in the present study

Therapeutic plasma % protein-unbound Concentration used References

concentration fraction in this study _______________________________________________________________________________________________________________

Dofetilide 1.4-18.4 ng/ml 30-40% 10 nM [22]

(or 3-46 nM)

Quinidine 2-5 μg/ml 20-30% 5 μM [23]

(or 6-15 μM)

Procainamide 4-8 μg/ml 60-65% 10 μM [24]

(or 15-30 μM)

Flecainide 0.2-1.0 μg/ml 60% 1.5 μM [25]

(or 0.5-2.5 μM)
